# Role of Rac1 GTPase in NADPH Oxidase Activation and Cognitive Impairment Following Cerebral Ischemia in the Rat

**DOI:** 10.1371/journal.pone.0012606

**Published:** 2010-09-07

**Authors:** Limor Raz, Quan-Guang Zhang, Cai-feng Zhou, Dong Han, Priya Gulati, Li-cai Yang, Fang Yang, Rui-min Wang, Darrell W. Brann

**Affiliations:** 1 Institute of Molecular Medicine and Genetics, Medical College of Georgia, Augusta, Georgia, United States of America; 2 Experimental and Research Center, North China Coal Medical University, Tangshan, Hebei, People's Republic of China; National Institutes of Health, United States of America

## Abstract

**Background:**

Recent work by our laboratory and others has implicated NADPH oxidase as having an important role in reactive oxygen species (ROS) generation and neuronal damage following cerebral ischemia, although the mechanisms controlling NADPH oxidase in the brain remain poorly understood. The purpose of the current study was to examine the regulatory and functional role of the Rho GTPase, Rac1 in NADPH oxidase activation, ROS generation and neuronal cell death/cognitive dysfunction following global cerebral ischemia in the male rat.

**Methodology/Principal Findings:**

Our studies revealed that NADPH oxidase activity and superoxide (O_2_^−^) production in the hippocampal CA1 region increased rapidly after cerebral ischemia to reach a peak at 3 h post-reperfusion, followed by a fall in levels by 24 h post-reperfusion. Administration of a Rac GTPase inhibitor (NSC23766) 15 min before cerebral ischemia significantly attenuated NADPH oxidase activation and O_2_^−^ production at 3 h after stroke as compared to vehicle-treated controls. NSC23766 also attenuated “*in situ*” O_2_^−^ production in the hippocampus after ischemia/reperfusion, as determined by fluorescent oxidized hydroethidine staining. Oxidative stress damage in the hippocampal CA1 after ischemia/reperfusion was also significantly attenuated by NSC23766 treatment, as evidenced by a marked attenuation of immunostaining for the oxidative stress damage markers, 4-HNE, 8-OHdG and H2AX at 24 h in the hippocampal CA1 region following cerebral ischemia. In addition, Morris Water maze testing revealed that Rac GTPase inhibition after ischemic injury significantly improved hippocampal-dependent memory and cognitive spatial abilities at 7–9 d post reperfusion as compared to vehicle-treated animals.

**Conclusions/Significance:**

The results of the study suggest that Rac1 GTPase has a critical role in mediating ischemia/reperfusion injury-induced NADPH oxidase activation, ROS generation and oxidative stress in the hippocampal CA1 region of the rat, and thus contributes significantly to neuronal degeneration and cognitive dysfunction following cerebral ischemia.

## Introduction

Ischemic stroke is a significant clinical problem of complex neuropathology, and is the third leading cause of death in the United States of America. Abundant evidence suggests that oxidative stress contributes significantly to the neuronal cell death that occurs following ischemic stroke. For instance, following reperfusion of the brain, there is a rapid increase of reactive oxygen species (ROS) and reactive nitrogen species (RNS), which has been shown to result in increased lipid peroxidation, protein nitrosylation, DNA damage and blood brain barrier dysfunction, thereby contributing to neuronal cell death [1]–[3]. It has been previously suggested that mitochondria are a primary source of ROS following ischemic reperfusion [4], [5]. Recently however, there is emerging evidence that a membrane enzyme, NADPH oxidase, contributes significantly to ROS generation following ischemic reperfusion [6]–[8]. Activation of NADPH oxidase leads to generation of the superoxide ion (O_2_^−^), a ROS which can be converted to the highly reactive hydroxyl radical and to peroxynitrite, a highly damaging RNS [2], [8]. To date, five NADPH oxidase enzyme (NOX) isoforms have been identified (NOX 1–5), and localization studies have shown that of the five NOX enzymes, the NOX2 and NOX4 isoforms are highly localized in the hippocampus CA1 and cerebral cortex [9], [10]. NOX2 appears particularly important in oxidative stress-induced neuronal cell death following stroke, as evidenced by a dramatic reduction in infarct size in NOX2 knockout animals [11], and in NOX2 inhibitor-treated animals [12].

The factors that regulate brain NOX2 NADPH oxidase activation are not well understood. Studies in immune cells have provided evidence that the Rho GTPase, Rac1, plays an important role in NOX2 activation in immune cells. Upon cellular stress, Rac dissociates from its cytosolic complex with guanosine diphosphate (GDP) dissociation inhibitor (GDI). The inactive Rac-GDP is then exchanged for GTP through the action of guanine nucleotide exchange factors (GEFs), Tiam1 and Trio, which are located on the membrane. Once active, Rac-GTP associates with the membrane and interacts with the tetratricopeptide repeat (TPR) site on p67phox subunit [13] and with membrane bound NOX2, resulting in NADPH oxidase complex activation [14], [15]. Surprisingly, there is not much known on the role of Rac1 GTPase in the ischemic brain. Work from our group has shown that administration of the Rac GTPase inhibitor (NSC23766) decreases activation of the proapoptotic signaling kinase, JNK in the hippocampus CA1 region following ischemic stroke reperfusion and reduces apoptosis [7]. Furthermore, the hormone estrogen was shown to suppress Rac1 GTPase activation in the hippocampus following ischemic stroke as a component of its neuroprotective actions [12]. It is currently unknown whether Rac1 GTPase plays an important role in NADPH oxidase activation, ROS generation and oxidative stress in the hippocampus following stroke, and whether inhibition of Rac GTPase activation might preserve cognitive function following cerebral ischemia. The current study addressed these important questions using a 4-vessel global cerebral ischemia rat model, which induces significant damage to the hippocampus CA1 region, an area critical for memory and cognitive function.

## Methods

### Induction of global cerebral ischemia

The experimental model of global cerebral ischemia (GCI) has been widely used to study delayed neuronal cell death of sensitive hippocampus CA1 pyramidal neurons, which occurs 2–4 days after the onset of reperfusion [16]. In the current study, three-month old adult male Sprague–Dawley rats were used and GCI was induced by four-vessel occlusion, as described previously [17], [18]. Briefly, under anesthesia with chloral hydrate (350 mg/kg, i.p.), the vertebral arteries were electrocauterized and the common carotid arteries (CCA) were exposed. Wound clips were used to close the incision and the rats were allowed a 24 h recovery period. After 24 h, the animals were anesthetized using 3% isoflurane anesthesia and the CCA were re-exposed and clipped via aneurysm clips. A 10-min occlusion period was used for the GCI. Rats which lost their righting reflex within 30 sec and whose pupils were dilated and unresponsive to light during ischemia were selected for the experiments. Carotid artery blood flow was restored by releasing the clips allowing reperfusion. Rectal temperature was maintained at 37° Celsius using a thermal blanket throughout the experiment and 2 h post-ischemia. Sham controls underwent the same surgical exposure procedures, except that the arteries were not occluded. All procedures were approved by the Medical College of Georgia institutional committee for care and use of animals (#07-04-932) and were in accordance with National Institutes of Health guidelines.

### Histology analysis

Histological examination of the ischemic brain was performed by neuronal-specific nuclear protein (NeuN) and the neuronal degeneration marker (Fluoro-Jade B), as described previously by our laboratory [19]. Briefly, after perfusion with 0.9% saline followed by 4% paraformaldehyde (PFA) in 0.1 M phosphate buffer, the brains were post-fixed, cryoprotected with 30% sucrose until they sank, and frozen sectioned (20 µm) in the coronal plane of the dorsal hippocampus (∼2.5–4.5 mm posterior from bregma). Every fifth section was collected and used for staining. Staining for NeuN and Fluoro-Jade B was performed using a mouse anti-NeuN monoclonal antibody (1∶500; Millipore, Billerica, MA USA) and Fluoro-Jade B (AG310; Millipore). Images were captured on an LSM510 Meta confocal laser microscope (Carl Zeiss, Peabody, MA USA). Cells that positively stained with NeuN and negatively stained with Fluoro-Jade B were identified as “surviving neurons”; in contrast, double-stained yellow-colored cells represent CA1 neurons undergoing degeneration.

### TUNEL staining

Terminal deoxynucleotidyl transferase-mediated biotinylated UTP nick end (TUNEL) staining was performed on the free-floating coronal sections using the In Situ Cell Death Detection kit (Roche, Basel, Switzerland) following the instructions of the manufacturer. Briefly, after washing with 0.1% PBS–Triton X-100, the slides were permeabilized with 10 µg/mL proteinase K in 10 mM Tris/HCl, pH 7.4, for 15 min and incubated with TUNEL reaction mixture, including enzyme solution (terminal deoxynucleotidyl transferase) and tetramethylrhodamine-labeled TUNEL-positive nucleotides in a humidified chamber for 1 h at 37° Celsius. Slides for negative control were incubated with the label solution without terminal transferase for TUNEL. Samples were analyzed with a LSM510 Meta confocal microscope. For quantitative analyses, the number of surviving neurons and TUNEL-positive cells per 250 µm length of medial CA1 pyramidal cell layer were counted bilaterally in four to five sections per animal to provide a single value for each animal. A mean ±SE was calculated from the data in each group, and statistical analysis was performed as described below.

### DAB staining

For DAB staining, sections were incubated with 10% normal donkey serum in PBS containing 0.1% Triton X-100 and 0.3% H2O2 for 1 h at room temperature to block nonspecific surfaces. Sections were then incubated with the primary antibodies overnight at 4° Celsius in PBS containing 0.1% Triton X-100. The antibodies used were as follows: mouse anti-4-hydroxy-2-nonenal (4-HNE) (1∶500; Genox, Baltimore, MD USA), mouse anti-8-hydroxy-2′-deoxyguanosine (8-OHdG) (1∶100; Genox), rabbit anti-p-H2A.X (1∶200; Cell Signaling Technology, Danvers, MA USA). Afterwards, sections were washed with the same buffer, followed by incubation with secondary biotinylated goat anti-mouse or goat anti-rabbit antibodies (Vector Laboratories, Burlingame, CA USA) at a dilution of 1∶200 in PBS containing 0.1% Triton X-100 for 1 h at room temperature. Sections were then washed, followed by incubation with ABC reagents for 1 h at room temperature in the same buffer. Sections were rinsed in the same buffer and incubated with DAB reagent according to the instructions of the manufacturer (Vector Laboratories) for 2–10 min. After DAB incubation, sections were briefly washed with distilled water and dehydrated in graded alcohols, cleared in xylene, and mounted using xylene-based mounting medium. Images were captured on an Axiophot-2 visible/fluorescence microscope using an AxioVision4Ac software system (Carl Zeiss).

### Confocal microscopy and image analysis

Images were captured on an LSM510 Meta confocal laser microscope (Carl Zeiss) using either a 5X or 40X oil-immersion Neofluor objective (1.3 numerical aperture) with the image size set at 1024×1024 pixels. The following excitation/emission laser filter settings were used for various chromophores: an argon/2 laser was used for Alexa-Fluor488, with excitation maximum at 490 nm and emission in the range of 505–530 nm, a helium–neon laser was used for Alexa-Fluor594 with excitation maximum at 543 nm and emission in the range of 568–615 nm, and a second helium–neon laser was used for Alexa-Fluor647 with excitation maximum at 633 nm and emission in the range of 650–800 nm. The captured images were viewed and analyzed using LSM510 Meta imaging software as previously described [20].

### *In situ* detection of superoxide production

The production of superoxide (O_2_^−^) free radicals was investigated using hydroethidine (HEt) (Invitrogen, Carlsbad, CA USA) as described previously by our group and others [12], [20], [21]. In the present study, HEt (1 mg/ml in 200 µl of PBS) was administered intravenously 30 min before ischemia. Animals were anesthetized using isoflurane 3 h after ischemia and transcardially perfused with cold PBS and 4% PFA. Sham non-ischemic control animals were also treated with HEt solution as O_2_^−^ production control. Fluorescent intensity of the oxidized HEt was measured on a confocal laser microscope using an excitation wavelength of 543 nm, and the emission was recorded at wavelengths between 560 and 590 nm. The images were then examined using LSM 510 image software.

### Brain Homogenates

For brain tissue preparation, rats were sacrificed under isoflurane anesthesia at 30 min, 3 h, 6 h and 24 h after GCI. The hippocampal CA1 region was micro-dissected from both sides of the hippocampal fissure and immediately frozen in liquid nitrogen. Tissues were homogenized with a Teflon-glass homogenizer in ice cold homogenization medium consisting of 50 mM HEPES (pH 7.4), 150 mM NaCl, 12 mM β-glycerophosphate, 3 mM dithiotheitol (DTT), 2 mM sodium orthovanadate (Na3VO4), 1 mM EGTA, 1 mM NaF, 1 mM phenylmethylsulfonyl fluoride (PMSF), 1% Triton X-100, and 10 µg/ml each of aprotinin, leupeptin, and pepstatin A. The homogenates were centrifuged at 15,000 ×g for 30 min at 4° Celsius, supernatants were collected and stored at −80° Celsius for use. The protein concentrations were determined by a Lowry protein assay kit with bovine serum albumin as standard.

### NADPH oxidase activity and superoxide production assay

NADPH oxidase activity was determined based on superoxide-induced lucigenin photoemissions, as described previously by our laboratory [12]. For assaying NADPH oxidase enzymatic activity, 50 µg of total fraction was used. Enzyme assays were performed in a final volume of 1 ml containing 50 mMKrebs'–Ringer's phosphate buffer, pH 7.0, 1 mM EGTA, 150 mM sucrose, 0.5 mM lucigenin, 0.1 mM NADPH, and tissue homogenate. Enzyme reactions were initiated with the addition of NADPH. No enzymatic activity could be detected in the absence of NADPH. Photoemissions, expressed in terms of relative light units (RLU), were measured every min for 5 min using a luminometer. Assays were performed in the dark at room temperature with appropriate controls. The rate of NADPH consumption was monitored by measuring the mean values in absorbance (340 nm), and NADPH oxidase activity was normalized by the amount of protein and the change in optical density (OD). Activity was calculated as OD per micrograms of protein per minute. Superoxide production was measured from total fractions using a LumiMax Superoxide Anion Detection kit (Stratagene, La Jolla, CA USA) following the protocol of the manufacturer. Briefly, 50 µg of sample protein was suspended in 100 µl of superoxide anion (SOA) assay medium and then mixed with 100 µl of reagent mixture containing 0.2 mM luminol and 0.25 mM enhancer in SOA assay medium. Light emissions at 30 sec intervals were recorded by a standard luminometer and absorbance was measured at 340 nm. Values were standardized to the amount of protein, and photons of light counted were expressed as RLU per microgram of protein. A mean ±SE was calculated from the data collected in each group for graphical depiction, expressed as fold change vs. sham control group. Statistical analysis of the data was performed as described below.

### Rac1-GTP binding assay

Rac1 activation assay was performed using PAK1-PBD color agarose beads (Cell Biolabs). Briefly, 400 µg samples were mixed with 20 µl of PAK1-PBD agarose beads and incubated for 1 h at 4° Celsius. The reaction was terminated by addition of MgCl_2_. The agarose beads were collected by spinning at 12,000×g for 1 min at 4° Celsius and the supernatants removed. Precipitated complexes were washed three times with magnesium-containing TBS buffer and boiled in sample buffer. Proteins were separated by 10% SDS-PAGE, transferred onto nitrocellulose membrane, and detected by immunoblotting using an anti-Rac1-specific antibody (1∶1000, BD Biosciences Transduction Laboratories).

### Intracerebroventricular administration

To investigate the role of Rac1 GTPase in response to ischemic injury, 25 µg of the Rac inhibitor, NSC23766 (Tocris Bioscience, Ellisville, Missouri, USA), was dissolved in 5 µL saline and administered bilaterally into the lateral cerebral ventricles of rats 15 min before ischemia for a total dose of 50 µg. Vehicle-treated control animals received bilateral saline injections. The 50 µg dose of the inhibitor was chosen based on a previous dose response curve performed in our lab which showed it to be the most optimal and effective dose in inhibiting Rac GTPase activation in the hippocampal CA1 region following intracerebroventricular (icv) injection, and which did not display any significant behavioral side effects [7]. To investigate the role of NOX2 NADPH oxidase, we utilized a specific competitive NOX2 inhibitor, gp91ds-tat, which is a 9 amino acid peptide sequence of the p47Phox docking site on NOX2 and prevents p47phox from forming a complex with NOX2 [22]. A scrambled gp91ds-tat peptide was used as a control. Gp91ds-tat and the scrambled peptide control was administered bilaterally via icv injection in the lateral ventricles at a dose of 100 ng in 5 µl. The dose of gp91ds-tat was chosen based on previous studies by our laboratory showing its effectiveness and lack of side effects [12]. For i.c.v. injections, the rats were placed on ear bars of a stereotaxic instrument under anesthesia. Drug infusion was performed using a steppermotorized microsyringe (Stoelting, Wood Dale, IL, USA) at a rate of 1 µL/min through a pre-implanted cannula in the cerebral ventricle (from the bregma: anteroposterior, ±0.8 mm; lateral, 1.5 mm; depth, 3.5 mm).

### Morris Water Maze

Rats were randomly assigned to the following groups: sham operated group (sham), NSC23766-treated sham group, ischemia/reperfusion group (I/R), NSC23766-treated group and I/R+vehicle group. On the 7^th^ day after ischemia onset, the Morris water maze test was performed to test spatial learning and memory as described previously by our laboratory and others [23]–[25]. The maze consisted of a black circular pool (diameter 2.14 m, height 80 cm, filled with water at 21–22° Celsius to a height of 50 cm). A black circular platform (9 cm in diameter) was placed 2 cm below the water line in the center of one quadrant, and remained in the same position. Several, constant, large visual cues surrounded the tank at a height of 120–150 cm to facilitate orientation. The rat was placed in the water facing the wall at one random start location out of four (north, south, east and west), located equidistance from one another around the rim of the pool. Each rat was then allowed to find the submerged platform within 90 sec, and rest on it for 20 sec. If the rat failed to find the hidden platform within allotted time, it was placed on it for 20 sec. The procedure was repeated for all the four start locations. The latency time, representing the average of the four trails to reach the platform and swimming speed were recorded. Two sessions of the four trails were conducted on the first testing day, within a 4 h interval. The first session was considered as a training procedure. One session out of the four trails was conducted daily on the next 2 testing days. Four hours after the last trail, a probe trail was performed within 90 sec, in which the platform was removed from the tank. The rat was placed in the water at the same random start location, and time spent in the quadrant of the pool, which previously contained the platform, was recorded. The test assessed how well the rats remembered the location of the hidden platform and whether the rats had a learned bias to navigate toward the goal quadrant.

### Statistical evaluation

Four to five animals were used per group. All values were expressed as the means ±SE. Statistical analysis of the results was carried out by one-way analysis of variance (ANOVA) followed by the Student-Newman–Keuls test. Statistical significance was accepted at the 95% confidence level. Differences of p<0.05 were considered significant.

## Results

### Rac1 GTPase activation contributes significantly to delayed neuronal cell degeneration in the hippocampus CA1 region following GCI

**Figure 1:**
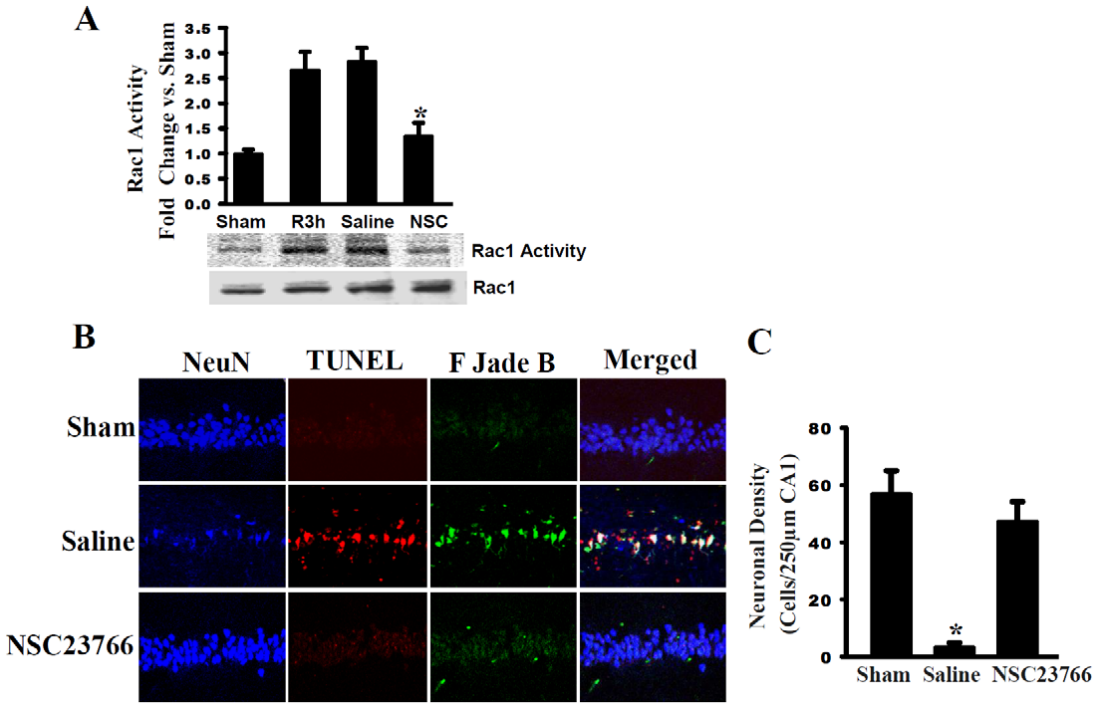
Rac GTPase Activation Contributes Significantly to Neuronal Cell Death in the Hippocampus CA1 Region Following Global Cerebral Ischemia (GCI). (**A**) NSC23766 significantly attenuated cerebral ischemia-induced Rac1 activity at 3 h after ischemia. *Represents P<0.05 (four to 5 animals) vs. reperfusion 3 hour (R3h) and saline treatment groups. (**B**) Representative hippocampal CA1 sections from sham, saline–treated (ischemia) and NSC23766-treated (ischemia) male rats were labeled with NeuN (Blue), TUNEL (Red) and FJadeB (Green) staining at 7 d following GCI. Merged images represent CA1 neurons undergoing degeneration, indicated by white staining. (**C**) Quantification showed that NSC23766 was strongly neuroprotective of CA1 region. Data was obtained from five independent animals and a typical experiment is presented. Results are expressed as neuronal density with mean ± SE. ^*^ *p*<0.05 vs. sham control and NSC23766.

**Figure 1** shows the results of the effect of administration of a Rac GTPase inhibitor, NSC23766 upon Rac1 GTPase activity (**Fig. 1A**) and neuronal cell survival (**Fig. 1B&C**) in the hippocampal CA1 region. As shown in **Figure 1A**, a PAK1 substrate was used to pull down active, GTP-bound Rac1 via its PBD domain, in order to examine Rac1 activity in total CA1 lysates at 3 h following ischemia/reperfusion. Our results show low, baseline Rac1 activity levels in sham non-ischemic animals, while there was a robust elevation of Rac1 activity in ischemic animals at 3 h post-reperfusion (R3h). Furthermore, saline treatment had no effect upon Rac1 activity at 3 h post-reperfusion, whereas treatment with the Rac GTPase inhibitor, NSC23766 resulted in ∼50% reduction in Rac1 activity as compared to controls. Note that total Rac1 protein expression was unchanged by any treatment. Data was reported in fold change versus sham, in 4–5 animals per treatment group. Taken altogether, our findings suggest NSC23766 to be an effective Rac1 inhibitor, having the ability to counteract Rac1 activation at 3 h following cerebral ischemia. **Figure 1B** shows that NSC23766 pretreatment significantly protected against delayed neuronal cell degeneration and death in the hippocampal CA1 region at 7 d after GCI. Representative photomicrographs of stained hippocampal CA1 sections revealed that GCI induced a significant loss of hippocampal CA1 neurons at 7 d following ischemic reperfusion, as indicated by a significant decrease in NeuN-positive stained cells in saline-treated animals following GCI as compared to sham controls (**Fig. 1B**). In addition, there was a significant increase in staining for Fluoro-Jade B (a neurodegeneration marker) and TUNEL (a method for detecting apoptosis) in the hippocampal CA1 region in saline-treated (ischemia) animals as compared to sham controls, which suggests there is increased neuronal degeneration and apoptosis following ischemic reperfusion. Merged, colocalized images of the hippocampus CA1 further demonstrate that the Fluoro-Jade B and TUNEL staining is predominantly *neuron*-specific. Intriguingly, icv administration of the Rac GTPase inhibitor (NSC23766) strongly protected the hippocampal CA1 neurons from neuronal degeneration and apoptotic cell death, as evidenced by preserved NeuN neuronal staining and strongly attenuated Fluoro-Jade B and TUNEL staining in NSC23766-treated animals as compared to saline controls (Figure 1B). In **Figure 1C**, quantification of staining results from all animals is provided (expressed in terms of neuronal density - *e.g.* number of hippocampal CA1 cells per 250 µm). Surviving neurons within the hippocampus CA1 are defined as NeuN-positive and Fluoro-Jade B- and TUNEL-negative stained cells. The results demonstrate a profound reduction in neuronal density in vehicle-treated ischemic animals as compared to sham (non-ischemic) animals. NSC23766 treatment markedly attenuated hippocampal CA1 neuronal death following GCI, suggesting a critical role for Rac GTPase activation in delayed neuronal cell degeneration and death in the hippocampal CA1 region following GCI.

### Temporal pattern of NADPH oxidase activation and superoxide (O_2_^−^) production in the rat hippocampal CA1 region following GCI

**Figure 2:**
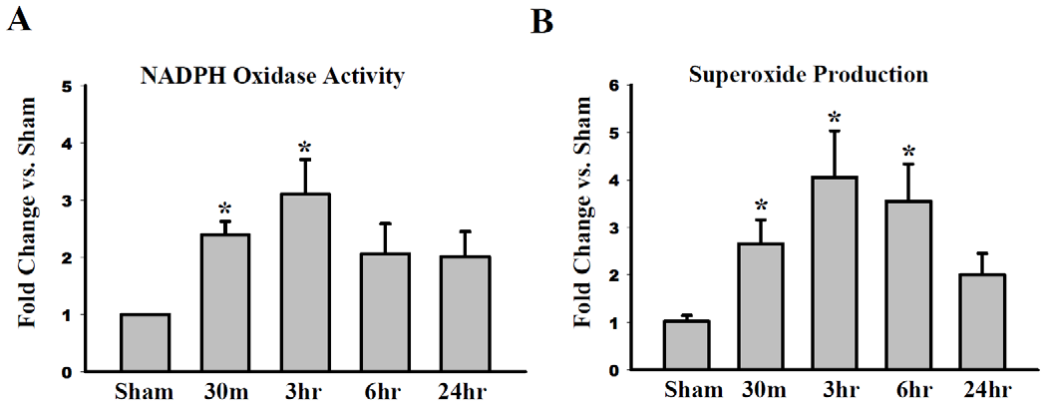
Temporal Expression of NADPH Oxidase Activity and Superoxide Production in the Hippocampal CA1 Region Following GCI. Homogenates taken from the hippocampus CA1 at 30 min, 3 h, 6 h, 24 h after reperfusion were subjected to NADPH oxidase activity (**A**) and superoxide production (**B**) assays to assess temporal expression following ischemia-reperfusion. Data is expressed as optical density (OD) and represented as fold vs. sham from four to five animals. ^*^ *p*<0.05 vs. sham control.

Rac1 GTPase activation has been suggested to be critical for NADPH oxidase activation in immune cells and may have a similar role in the brain. To explore this possibility, we first examined the temporal pattern of NADPH oxidase activation and O_2_^−^ production in the male rat hippocampal CA1 region following GCI. As shown in **Figure 2A**, NADPH oxidase activation in the hippocampal CA1 region increased rapidly following ischemic reperfusion, with an elevation observed as early as 30 min post-reperfusion, and peak NADPH oxidase activation levels observed at 3 h post-reperfusion (∼3 fold increase versus sham controls). NADPH oxidase activation levels subsequently fell at 6 h and 24 h after reperfusion, but mean levels were still higher than sham controls. Similarly, in **Figure 2B**, O_2_^−^ levels showed a similar rapid elevation at 30 min following GCI reperfusion, with peak levels observed at 3 h post-reperfusion (∼4-fold increase as compared to sham controls). Thereafter, O_2_^−^ levels fell slightly at 6 h post-reperfusion, and by 24 h post-reperfusion, O_2_^−^ levels, while still moderately elevated, were no longer statistically different from sham controls.

### Rac GTPase activation is critical for enhanced NADPH oxidase activity and O_2_^−^ production in the hippocampal CA1 region following GCI

**Figure 3:**
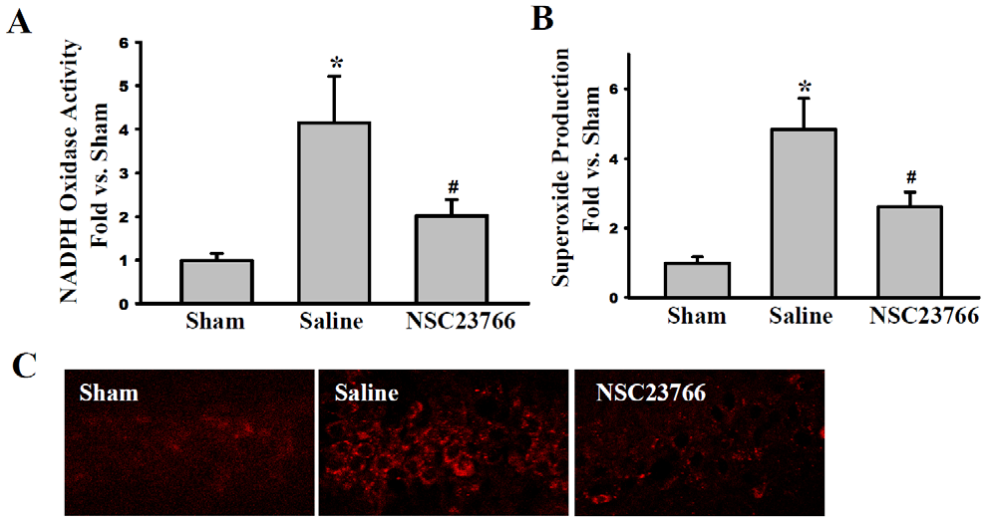
Rac GTPase Activation is Critical for Enhanced NADPH Oxidase Activity and Superoxide Production in the Hippocampal CA1 Region Following GCI. NSC23766 pre-treatment reduces NADPH oxidase activity (**A**) and superoxide production (**B**) at 3 h post-reperfusion in the hippocampus CA1 after GCI. Data for experiments (A) and (B) was measured as optical density (OD) and expressed as fold difference in comparison to sham from four to five animals. *p<0.05 vs. sham, ^#^ *p*<0.05 vs. saline control. HET staining measuring endogenous superoxide production in CA1 coronal brain sections obtained from four to five animals; magnification 40X (**C**).

We next examined the role of Rac GTPase activation in enhanced NADPH oxidase activation and O_2_^−^ production in the hippocampal CA1 region following GCI by determining the effect of inhibiting Rac GTPase activation via administration of NSC23766. NADPH oxidase activation and O_2_^−^ production were examined at 3 h following ischemia-reperfusion (e.g. the time-point of peak NADPH oxidase activation and O_2_^−^ production following GCI), and *in situ* O_2_^−^ production was also assessed using the *in situ* oxidized hydroethidine (HEt) method, in which HEt, a marker of O_2_^−^ production, is selectively taken up by cells and oxidized by O_2_^−^ into ethidium, which provides a red fluorescence signal. As shown in **Figure 3A–B**, treatment with NSC23766 significantly attenuated the GCI-induced elevation of NADPH oxidase activation and O_2_^−^ production in the hippocampal CA1 region at 3 h after reperfusion. In addition, “in situ” O_2_^−^ production, as measured by oxidized HEt staining, confirmed increased O_2_^−^ production in the hippocampal CA1 region following GCI, which was strongly attenuated by the Rac inhibitor, NSC23766 (**Figure 3C**).

### Contribution of NADPH Oxidase to Ischemic Neuronal Cell Death at 7d Post-Reperfusion, in the Hippocampal CA1 Region

**Figure 4:**
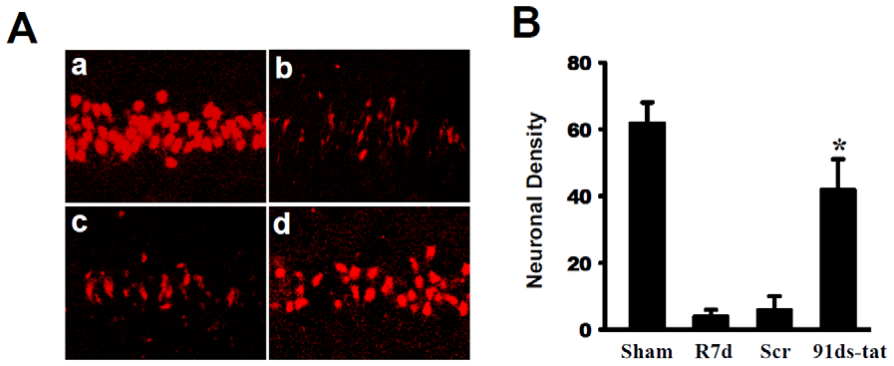
Contributions of NADPH oxidase to Ischemic Neuronal Damage in Hippocampal CA1. (**A**) Representative staining of coronal CA1 sections with NeuN (red) show neuroprotection of gp91ds-tat peptide at day 7 after reperfusion. a: Sham; b: Ischemic reperfusion; c: Scrambled gp91ds-tat control; d: gp91ds-tat (91ds-tat). (**B**) CA1 neuronal density was counted per 250 µm length of medial CA1 region from five to six rats in each group. *p<0.05 vs. reperfusion at 7 days (R7d) and Scr treatment groups. Scr: gp91ds-tat control.

To confirm the critical role of NADPH oxidase complex formation and in GCI-induced neuronal cell death, we injected male rats with a specific competitive NOX2 inhibitor, gp91ds-tat (100 ng bilaterally icv, 30 min prior to GCI). In addition, a scrambled tat peptide which lacks NADPH oxidase inhibitory activity was used as a control. Coronal CA1 sections were collected at 7 d after GCI and stained with NeuN neuronal marker to determine neuronal survival following GCI. As shown in **Fig. 4A**, the results revealed that NeuN staining was high in the hippocampal CA1 region in sham (non-ischemic) animals (Fig. 4A, panel a), while ischemia/reperfusion and scrambled-tat injected control groups (Fig. 4A, panels b,c, respectively) showed a distinct loss of NeuN-stained neurons in the CA1 region 7 d after ischemia. In contrast, gp91ds-tat administration exerted significant neuroprotection, as indicated by preservation of NeuN-positive neurons in the hippocampal CA1 region (Fig. 4A, panel d). Quantification of the results is provided in **Figure 4B** which demonstrates a neuroprotective effect of gp91ds-tat. As a whole, these findings suggest that NADPH oxidase activation plays a significant role in neuronal cell death in the hippocampal CA1 region following GCI.

### Inhibition of Rac GTPase activation attenuates induction of oxidative stress damage in the hippocampus CA1 after GCI

**Figure 5:**
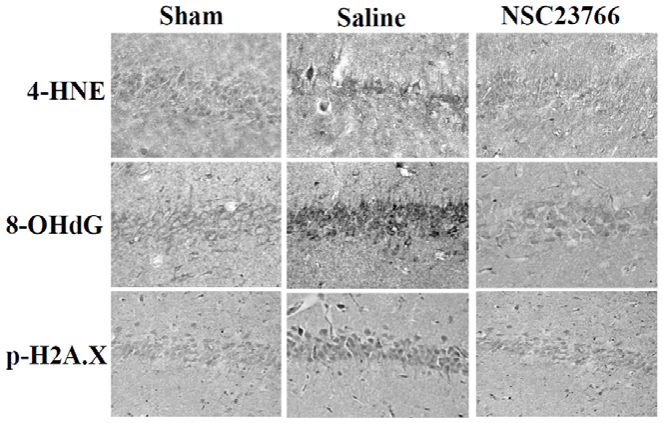
Rac GTPase Activation is Critical for Enhanced Oxidative Damage in the Hippocampus CA1 Following GCI. DAB staining of representative coronal CA1 sections show NSC23766 ability to attenuate staining for oxidative stress markers for lipid peroxidation (4-HNE), DNA damage (8-OHdG) and histone phosphorylation (p-H2A.X). (Four to five animals per treatment group, magnification used was 20X).

We next examined the effect of Rac GTPase inhibition upon oxidative stress damage in the hippocampal CA1 region following GCI. Immunostaining for the oxidative stress markers, 4-HNE (4-hydroxynonenal), 8-OHdG (8-hydroxydeoxyguanosine), and p-H2AX (phospho-histone) was conducted at 24 h after GCI to assess lipid peroxidation, DNA damage and oxidative histone phosphorylation, respectively. The results show robust increases in immunostaining intensity for all three oxidative stress markers in the saline-injected (ischemia) group as compared to sham controls (**Figure 5**). Rac GTPase inhibition through administration of NSC23766 resulted in an almost complete attenuation of the oxidative stress damage as indicated by a strong attenuation of 4-HNE, 8-OHdG and p-H2AX immunostaining in the hippocampal CA1 region as compared to saline (ischemia) controls.

### Functional outcome of Rac GTPase inhibition on learning and spatial memory in the hippocampal CA1 region

**Figure 6:**
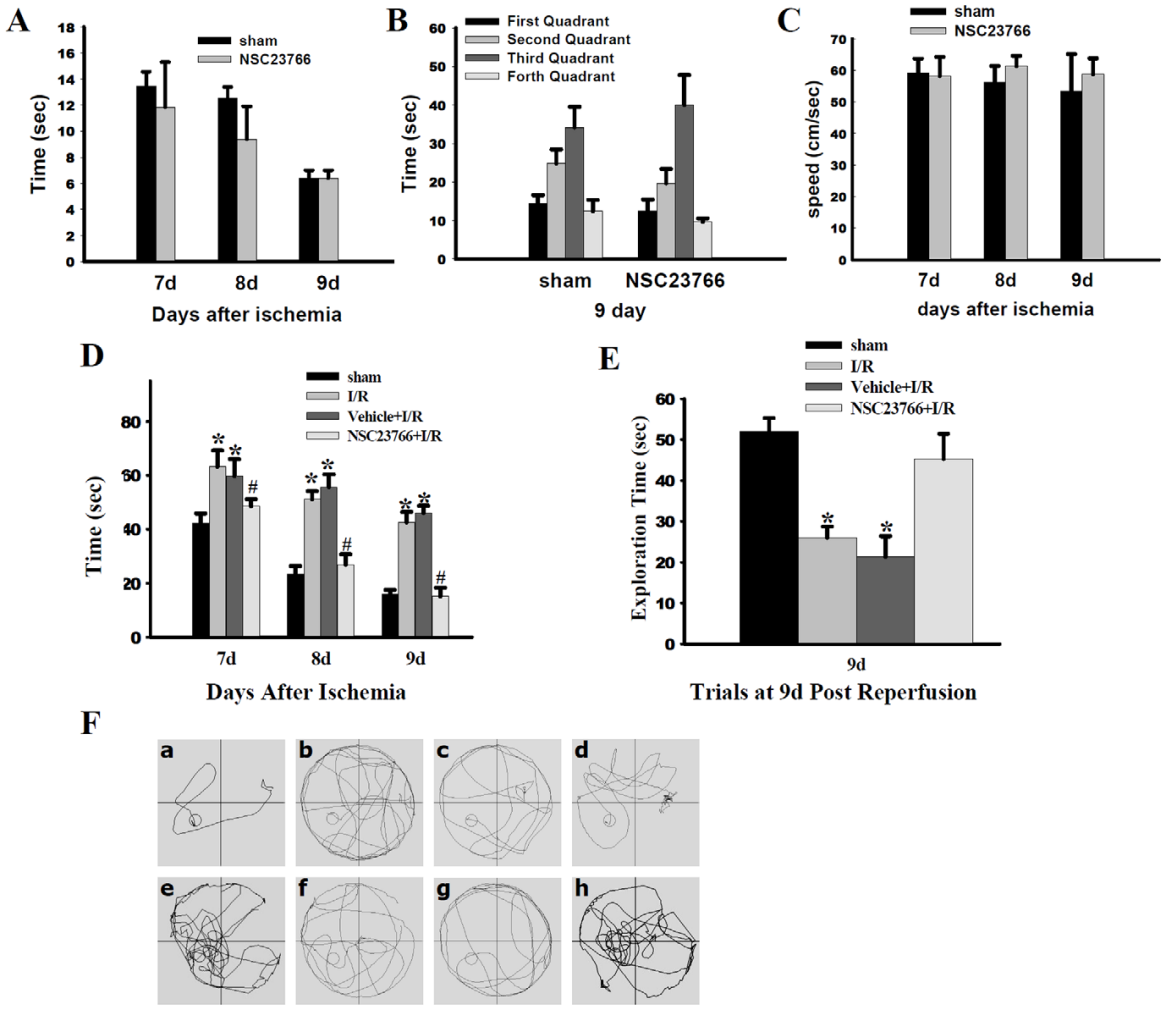
Effect of the Rac GTPase Inhibitor, NSC23766 Upon Cognitive Function Following GCI. Latency trial (**A**) and probe trial (**B**) results in Morris water maze of non-ischemic control sham and NSC23766 animals showing no effect of NSC23766 in nonischemic control animals. Panel **C** shows no significant differences between sham and NSC23766-treated animals in swimming speed (cm/sec); Five animals per condition, mean ± SE. (**D**) Time (sec) spent finding the submerged platform at 7 d, 8 d and 9 d after ischemic injury. (**E**) Exploration time spent in the quadrant which initially contained the platform at 9 d following reperfusion. (**F**) Representative traces indicating the sample paths of the rats from the maze latency trials (*a–d*) and the swimming traces from probe trials (*e–h*) (a, e: sham; b, f: I/R; c, g: vehicle + I/R; d, h: NSC23766 + I/R). Data is expressed as mean ± SE from five different animals.*p<0.05 vs. sham, ^#^ *p*<0.05 vs. vehicle + I/R. I: ischemia; R: reperfusion.

To determine the functional importance and role of Rac GTPase activation in cognitive decline following GCI, the Morris water maze test was used to assess spatial navigation on a spatial memory at 7–9 days after ischemic reperfusion. In **Figure 6A** and **6B**, we first compared sham non-ischemic animals' performance in the Morris water maze to that of Rac inhibitor treated rats at 7 d, 8 d and 9 d post-reperfusion. Both probe and latency trials failed to show any significant difference between Rac inhibitor-treated (NSC23766) and vehicle (sham) control (non-ischemic) animals in locating the hidden platform or navigating towards the goal quadrant. Furthermore, **Fig. 6C** shows that NSC23766 treatment did not affect swimming speed at 7 d, 8 d and 9 d after GCI. **Figure 6D** illustrates NSC23766 effects on latency time in finding the hidden platform during probe trials. There was no significant difference between ischemic rats (I/R) and saline-injected (Vehicle + I/R) animals in finding the submerged platform. In contrast, sham-operated and NSC23766-treated rats' latency time became progressively shorter in a day-dependent manner. On day one, both I/R and vehicle + I/R animals spent a longer time searching for the hidden platform, as compared to sham. Interestingly, NSC23766-treated rats exhibited shorter exploration times, marked by the rapid discovery of the hidden platform's location, yet no statistically significant differences were present between NSC23766 and vehicle-injected animals. On the second testing day, however, NSC23766-treated animals showed a latency time to finding the hidden platform similar to sham-operated rats, and displayed a profoundly shorter latency time as compared to the vehicle + I/R group. Along the same lines, the last day of the probed trials revealed a similar pattern, where rats in sham and NSC23766-treated groups spent a considerably shorter time in finding the hidden platform than vehicle-treated group. Representative trace diagrams indicating the latency time to finding the submerged platform are depicted in **Figure 6F**, panels a, b, c, d. In probe trails characterized by the removal of the hidden platform (**Figure 6E**), ischemic and vehicle-treated rats displayed worse learned bias navigating towards the goal quadrant, which previously contained the platform. They spent less time in the goal quadrant than their sham counterparts. NSC23766-treated rats, on the other hand, displayed improved learned bias, as evidenced by spending more time in the goal quadrant than ischemic animals. In fact, there was no statistical difference between NSC23766 and sham animals in the time spent searching for the hidden platform. It should be added that differences in the swimming speed were negligible between the various experimental groups (data not shown, *p*>0.05). Representative traces obtained during the specified probed trials are depicted in **Figure 6F** - e, f, g, h. Overall, these results suggest that Rac GTPase activation plays an important pathological role in ischemic stress-induced cognitive impairment.

## Discussion

The current study adds to growing evidence of an important role of Rac1 GTPase in oxidative stress and neuronal cell death following cerebral ischemia. Previous work by our group demonstrated that Rac1 GTPase activation increases from 10 min to 72 h in the hippocampal CA1 region following GCI in rats, with peak levels from 3 h–6 h post reperfusion [7], [12]. The elevation of Rac GTPase activation appears to contribute to the pathology following ischemic stress as evidenced by our finding that administration of a Rac GTPase inhibitor, NSC23766 or Rac1 antisense oligonucleotides [7] attenuated apoptotic neuronal cell death following GCI. In addition, the current study revealed that neuronal degeneration in the hippocampal CA1 region was also markedly inhibited by the pretreatment of the Rac GTPase inhibitor following GCI, suggesting an important role of Rac GTPase activation in delayed neuronal degeneration following ischemia/reperfusion injury. It should be mentioned that we also examined post-treatment administration of NSC23766 (15 min. after ischemia; 25 µg bilaterally i.c.v.) and while we found it to enhance survival of hippocampal neurons (29.25±4.48) as compared to saline-treated controls (4.25±1.25), the effect was not as robust as that observed with pretreatment (data not shown).

Our study also provides insight into how Rac1 GTPase activation contributes to cerebral ischemia pathology, by demonstrating that Rac1 GTPase activation is critical for induction of oxidative stress in the hippocampal CA1 region following ischemia/reperfusion injury. Inhibition of Rac GTPase activation markedly decreased oxidative neuronal damage to hippocampal CA1 region lipids, proteins, and DNA following ischemia/reperfusion injury. The decrease in oxidative stress following Rac GTPase inhibition is likely due to a correlated significant attenuation of O_2_^−^ generation, which was observed following ischemia/reperfusion. Interestingly, targeted inhibition of Rac1 GTPase via a dominant negative approach has been shown to similarly exert anti-oxidative stress effects against ischemia/reperfusion injury in the liver [26]. Furthermore, deletion of Rac1 GTPase in mouse rod photoreceptors via a Cre/Lox approach has been shown to strongly protect rod photoreceptors against photo-oxidative stress [27]. Rac1 is also expressed in endothelium of vascular cells, and targeted deletion of endothelial Rac1 has been demonstrated to reduce focal cerebral ischemia-induced edema in mice [28]. Additionally, Kahles and colleagues have demonstrated that in *vivo* Rac1 inhibition via administration of the 3-hydroxy-3-methylglutaryl coenzyme A reductase inhibitor, atorvastatin, prevented ischemia/reperfusion-induced blood brain barrier disruption [3]. These findings, coupled with the results of the current study, support a critical role for Rac1 GTPase activation in the induction of oxidative stress in *both* neural and non-neural tissues.

Our results further suggest that Rac1 GTPase contributes to induction of oxidative stress in the hippocampus by facilitating activation of NADPH oxidase, a key membrane enzyme that generates O_2_^−^ ions [6], [12], [29]. NSC23766 strongly attenuated NADPH oxidase activation and O_2_^−^ generation in the hippocampal CA1 following GCI, as well as oxidized HEt staining, which is a marker of *in situ* O_2_^−^ generation. Moreover, we show that NADPH oxidase inhibition via the gp91ds-tat NOX2 inhibitor is neuroprotective of hippocampus CA1 pyramidal cells at 7 d after ischemia/reperfusion, further confirming the importance of NADPH oxidase in neuronal cell death in the hippocampus after global cerebral ischemia. Studies in phagocytic cells have shown that Rac1 GTPase plays a key role in activation of the NOX2 NADPH oxidase isoform by a direct binding and recruiting of the p67phox subunit to the membrane [13], [30]. Rac1 GTPase may also be involved in activation of NOX1 and NOX3 isoforms, but it does not appear to be involved in activation of the NOX4, NOX 5 or Duox isoforms [30]. Work by our lab and others has provided evidence that the NOX2 isoform is a major contributor to O_2_^−^ production and oxidative stress neuronal damage in the hippocampus and cerebral cortex following cerebral ischemia, as evidenced by studies using a competitive NOX2 inhibitor and NOX2 knockout mice [11], [12]. Based on these observations, it is proposed that Rac1 GTPase likely regulates O_2_^−^ generation and oxidative stress in the hippocampus following GCI through a regulatory action on the *NOX2* isoform in the brain. However, a regulatory effect by Rac1 GTPase on NOX1 and NOX3 in the brain cannot be entirely excluded and deserves further study.

A functionally important role of Rac GTPase activation in the pathology of cerebral ischemia was further suggested by our studies using the Morris Water Maze, which revealed preservation of spatial learning and memory in Rac GTPase inhibitor-treated animals as compared to vehicle-treated controls at 7–9 days post ischemia/reperfusion. Swim speed was not different between the groups, demonstrating that the differences were not due to differences in locomotor ability or coordination. The Morris Water Maze is a hippocampal-dependent test of learning and memory, and thus the enhanced performance of the Rac GTPase inhibitor-treated animals on the Morris Water Maze is likely reflective of the attenuation of oxidative stress and enhanced neuronal survival in the hippocampal CA1 region of NSC23766-treated rats as compared to vehicle-treated (ischemia) rats. Mechanistically speaking, NSC23766 has been shown to inhibit Rac1 binding and activation by preventing interaction of the Rac-specific guanine exchange factors (GEFs), *Trio* and *Tiam1*, with Rac1 [31]. Thus, it is postulated that regulation of Tiam1 and Trio following cerebral ischemia contributes to the enhanced Rac1 GTPase activation following ischemia/reperfusion. Additional studies are needed to address this interesting possibility.

Finally, it is likely that Rac1 GTPase contributes to *multiple* pathological events and signaling pathways, which collectively facilitate neuronal damage and cognitive dysfunction following cerebral ischemia. For instance, previous work by our group has shown that Rac1 GTPase also has an important role in activation of the proapoptotic JNK signaling pathway following cerebral ischemia, as Rac1 binds to a scaffold complex of POSH and MLK3 and facilitates activation of JNK [7], [32]. Thus, in addition to enhancing NADPH oxidase activation and oxidative stress following ischemia/reperfusion, Rac1 also enhances proapototic JNK signaling, which collectively can enhance oxidative damage and apoptotic neuronal cell death in the hippocampal CA1 region, leading to hippocampal dysfunction and cognitive decline.

In conclusion, the current study provides evidence of an important role of Rac1 GTPase in ischemia/reperfusion injury-induced NADPH oxidase activation, O_2_^−^ production and oxidative stress in the hippocampal CA1 region following GCI, and thereby contributes significantly to the delayed neuronal cell death and to a negative functional cognitive outcome following cerebral ischemia. Studies that target Rac1 GTPase for inhibition may thus have efficacy in preservation of neuronal survival and cognitive function following stroke in humans, and therefore should be further explored.
